# Mn_2_Ga_2_S_5_ and Mn_2_Al_2_Se_5_ van der Waals Chalcogenides: A Source of Atomically Thin Nanomaterials

**DOI:** 10.3390/molecules29092026

**Published:** 2024-04-28

**Authors:** Ivan V. Chernoukhov, Alexey V. Bogach, Kirill A. Cherednichenko, Ruslan A. Gashigullin, Andrei V. Shevelkov, Valeriy Yu. Verchenko

**Affiliations:** 1 Department of Chemistry, Lomonosov Moscow State University, 119991 Moscow, Russia; 2 Prokhorov General Physics Institute of the Russian Academy of Sciences, 119991 Moscow, Russia; 3 Department of Physical and Colloid Chemistry, Gubkin University, 119991 Moscow, Russia

**Keywords:** van der Waals chalcogenides, exfoliation, manganese, nanomaterials, frustrated magnetism

## Abstract

Layered chalcogenides containing 3d transition metals are promising for the development of two-dimensional nanomaterials with interesting magnetic properties. Both mechanical and solution-based exfoliation of atomically thin layers is possible due to the low-energy van der Waals bonds. In this paper, we present the synthesis and crystal structures of the Mn_2_Ga_2_S_5_ and Mn_2_Al_2_Se_5_ layered chalcogenides. For Mn_2_Ga_2_S_5_, we report magnetic properties, as well as the exfoliation of nanofilms and nanoscrolls. The synthesis of both polycrystalline phases and single crystals is described, and their chemical stability in air is studied. Crystal structures are probed via powder X-ray diffraction and high-resolution transmission electron microscopy. The new compound Mn_2_Al_2_Se_5_ is isomorphous with Mn_2_Ga_2_S_5_ crystallizing in the Mg_2_Al_2_Se_5_ structure type. The crystal structure is built by the ABCBCA sequence of hexagonal close-packing layers of chalcogen atoms, where Mn^2+^ and Al^3+^/Ga^3+^ species preferentially occupy octahedral and tetrahedral voids, respectively. Mn_2_Ga_2_S_5_ exhibits an antiferromagnetic-like transition at 13 K accompanied by the ferromagnetic hysteresis of magnetization. Significant frustration of the magnetic system may yield spin-glass behavior at low temperatures. The exfoliation of Mn_2_Ga_2_S_5_ layers was performed in a non-polar solvent. Nanolayers and nanoscrolls were observed using high-resolution transmission electron microscopy. Fragments of micron-sized crystallites with a thickness of 70–100 nanometers were deposited on a glass surface, as evidenced by atomic force microscopy.

## 1. Introduction

Layered compounds of transition metals demonstrate interesting functional properties. In such compounds, it is possible to change the chemical composition by various substitutions [1], including solid solutions [2], intercalations [3], or the formation of intergrowth structures [4]. All these possibilities can be used to significantly modify the electronic band structure, yielding the semiconducting [5], magnetic [3], and magnetoresistive properties [6]. Furthermore, the formation of ferromagnetic, antiferromagnetic, and helicoidal magnetic structures, the appearance of magnetoelectric and multiferroic properties, and the creation of frustrated low-dimensional systems is possible [7,8]. In the case of halide, chalcogenide, and pnictide systems, bulk materials can be exfoliated via a wet technique in liquids [9] or the simple separation of layers using an adhesive tape [10]. These techniques are easy to implement compared to molecular beam epitaxy or organometallic chemical vapor deposition. Theoretical studies indicate the possibility of creating spin field-effect transistors based on the chalcogenide materials [5]. Furthermore, two-dimensional materials may demonstrate the switching of the type of magnetic ordering with respect to the bulk material. For example, in the case of an A-type antiferromagnet, the exfoliated atomically thin materials have surprisingly show ferromagnetic properties [7]. Moreover, during the formation of heterostructures, more complex magnetic behavior can be obtained due to the edge states. For example, the switch between the symmetric and antisymmetric magnetoresistance effect was observed in the MnPS_3_/Fe_3_GeTe_2_ heterostructure [11]. Also, the heterostructures may show interesting properties in the vicinity of room temperature, including strong plasmon-exciton coupling [12]. These properties open a wide field for the creation of new micro- and nanodevices.

Mn-based layered chalcogenides are cutting-edge materials with peculiar magnetic and electronic properties in the two-dimensional limit. For example, MnPS_3_ is a source of nanomaterials with a thickness of several atomic layers and outstanding functional properties [13,14,15,16,17]. For this compound, the dielectric breakdown strength of *E* = 5.41 × 10^6^ V/cm at the tunneling barrier height of *φ* = 1.31 V (for 9 or more layers) was observed, which can be used in field-effect transistors or magnetic tunnel junctions [18]. In the case of MnBi_2_Te_4_ [19,20,21,22,23], an odd–even layer-number effect was observed for atomically thin materials, which can be applied in heterostructures too [24]. Recently, the MnAl_2_Se_4_ and MnAl_2_S_4_ layered chalcogenides attracted interest as a source of nanomaterials [25]. These compounds are candidates for frustrated magnetism and spin-glass behavior [26]. Their crystal structures are homologous to those of the Mg_2_Al_2_Se_5_ structure type [27], which fosters the search for related compounds.

In this paper, we present the Mn_2_Ga_2_S_5_ and Mn_2_Al_2_Se_5_ isomorphous layered chalcogenides as a promising source of two-dimensional materials. Mn_2_Al_2_Se_5_ is a new compound, and we report its synthesis and crystal structure for the first time. Mn_2_Ga_2_S_5_ was first detected during exploratory syntheses in the MnS-Ga_2_S_3_ system [28]. Recently, the crystal structure, magnetic properties, and heat capacity of Mn_2_Ga_2_S_5_ have been reported [29,30], indicating the significant spin-glass character of the magnetic system. Here, we report synthesis, crystal growth, magnetic properties, and exfoliation of Mn_2_Ga_2_S_5_. Together with Mn_2_Al_2_Se_5_, these layered chalcogenides postulate the new “225” family of Mn-based materials with interesting magnetic properties.

## 2. Results and Discussion

### 2.1. Synthesis and Air Stability

The Mn_2_Ga_2_S_5_ compound was synthesized by annealing the elements at 1173 K in evacuated quartz ampule. For the Mn_2_Al_2_Se_5_ compound, binary selenides should be pre-synthesized since the elemental precursors strongly react with a quartz ampule at high temperatures. The synthesis of MnSe and Al_2_Se_3_ proceeds at the lower temperature of 973 K. After that, they were weighed according to the stoichiometric composition, carefully ground, and pressed into a pellet. Synthesis of Mn_2_Al_2_Se_5_ was performed at 1123 K to reduce possible degradation of quartz ampule. All operations with samples were carried out in a glove box filled with argon because the intermediate and target compounds are capable of oxidizing/hydrolyzing in air.

**Figure 1:**
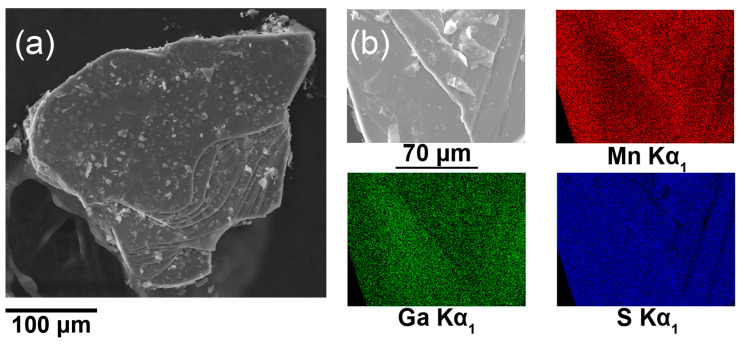
(**a**) Scanning electron microscopy (SEM) image of the Mn_2_Ga_2_S_5_ single crystal; (**b**) EDXS mapping of the surface.

The Mn_2_Ga_2_S_5_ single crystals were synthesized via chemical vapor transport reactions. For this purpose, various transport agents, such as S, I_2_, and HgI_2_, were tested. The highest quality crystals of the target phase were observed when using 15 mg HgI_2_ per 500 mg of the sample. The resulting mixture was sealed into an evacuated long quartz ampule and annealed in a furnace with a temperature gradient of ΔT = 100 K. The resulting mica-like crystals were submillimeter in size. The composition of the crystals, according to energy-dispersive X-ray spectroscopy (EDXS), is Mn_2_Ga_2.0(2)_S_5.5(4)_ and thus consistent with the nominal one. EDXS mapping shows the homogeneous distribution of Mn, Ga, and S (Figure 1).

The synthesis of nanomaterials was carried out on a glass substrate. The polycrystalline sample of Mn_2_Ga_2_S_5_ was placed in a heptane and was subjected to ultrasonic irradiation for 20 min at a temperature of 40 °C. The resulting colloidal solution was settled for 10 min and poured into a new container. Deposition onto the substrate was carried out at room temperature in small portions of 0.02 mL. The interval between depositions was 1 min.

**Figure 2:**
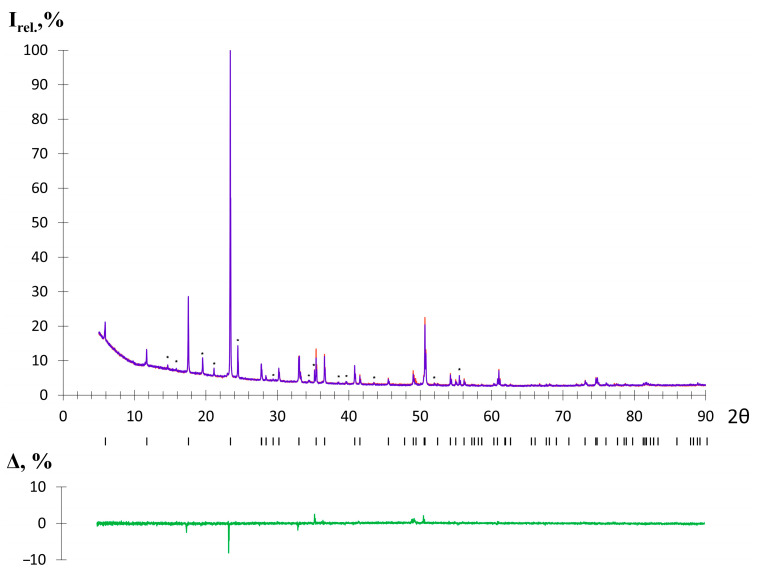
Chemical stability of Mn_2_Ga_2_S_5_ in air**.** PXRD patterns of Mn_2_Ga_2_S_5_ before and after reaction with air are shown as purple and orange lines, respectively. The positions of the peaks are shown by black ticks. The peaks of the SiO_2_ admixture are marked by asterisks. The difference curve is shown in green (I_max_ = 52,891 counts).

**Figure 3:**
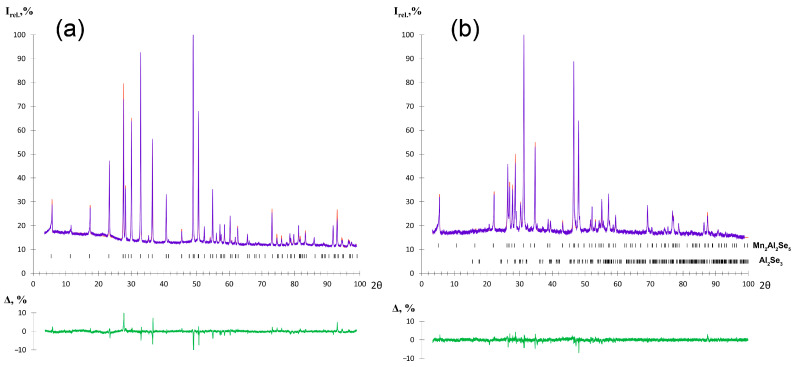
PXRD patterns of Mn_2_Ga_2_S_5_ (**a**) (I_max_ = 40,011 counts) and Mn_2_Al_2_Se_5_ (**b**) (I_max_ = 19,168 counts). The experimental data are shown by the purple dots, and the theoretical pattern is presented by the orange line. Black ticks show the positions of reflections. The difference curve is shown as a green line.

The chemical stability of Mn_2_Ga_2_S_5_ and Mn_2_Al_2_Se_5_ in air was checked using powder X-ray diffraction (PXRD). To do this, the samples were fixed on an open holder. PXRD patterns were registered immediately and after storing the samples in air. The signal accumulation time was identical in both PXRD experiments. Figure 2 shows that Mn_2_Ga_2_S_5_ is stable in air for a long time (1 week was tested). However, for the Mn_2_Al_2_Se_5_ sample, the broadening of reflections and the drop in intensity were already observed after 15 min in air. In addition, as soon as the Mn_2_Al_2_Se_5_ sample appears in the air, a characteristic odor of hydrogen selenide is detected. Notably, the sample contains a small admixture of Al_2_Se_3_ ~ 5 mass % (Figure 3), which is a strong Lewis acid. The hydrolysis reaction is presumably connected with the presence of Al_2_Se_3_, which is used as a starting material. Attempts to synthesize Mn_2_Al_2_Se_5_ without the admixture of Al_2_Se_3_ were not successful due to the formation of the MnAl_2_Se_4_ homologous compound [25].

### 2.2. Crystal Structure

**Figure 4:**
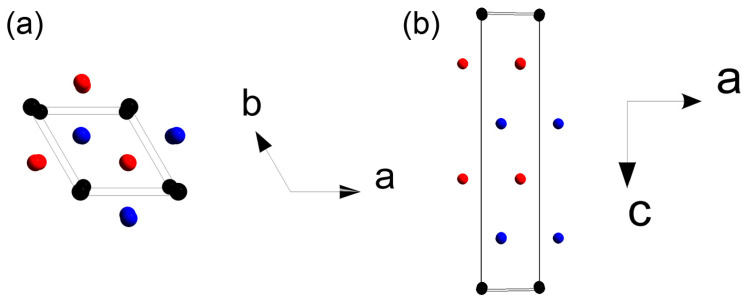
Densest hexagonal packing of Se atoms in the Mg_2_Al_2_Se_5_ structure: (**a**) View along the *c* direction; (**b**) view along the *b* direction. Black spheres indicate the A-plane of Se atoms, and blue and red indicate the B- and C-planes, respectively.

The Mn_2_Ga_2_S_5_ compound was obtained as a single-phase sample, and a small admixture of Al_2_Se_3_ was found in Mn_2_Al_2_Se_5_ according to PXRD (Figure 3). The crystal structure was refined by the Rietveld method using the Mg_2_Al_2_Se_5_ structure as a starting model [27]. CCDC 2344029 and 2344030 deposition numbers can be used to request the structural data. The measurement and refinement details, parameters of atomic positions, and selected interatomic distances are given in Table 1, Table 2 and Table 3, respectively. The Mg_2_Al_2_Se_5_ prototype compound crystallizes in the *P*$\bar{3}$*m*1 space group with unit cell parameters of *a* = 3.88 Å, *c* = 16.00 Å. The crystal structure can be described as the densest hexagonal packing of Se atoms, where the Se layers alternate in the order of (ABCBCA) (Figure 4). Octahedral voids are filled with magnesium cations between the layers A-B and C-A, while aluminum cations are located in tetrahedral voids between the layers B-C and B-C. The van der Waals gap is formed between the C-B layers in the middle of unit cell (in this interlayer space, the voids are not occupied by cations). In the initial structure, the mixed occupation of cationic positions is not described [27].

**Figure 5:**
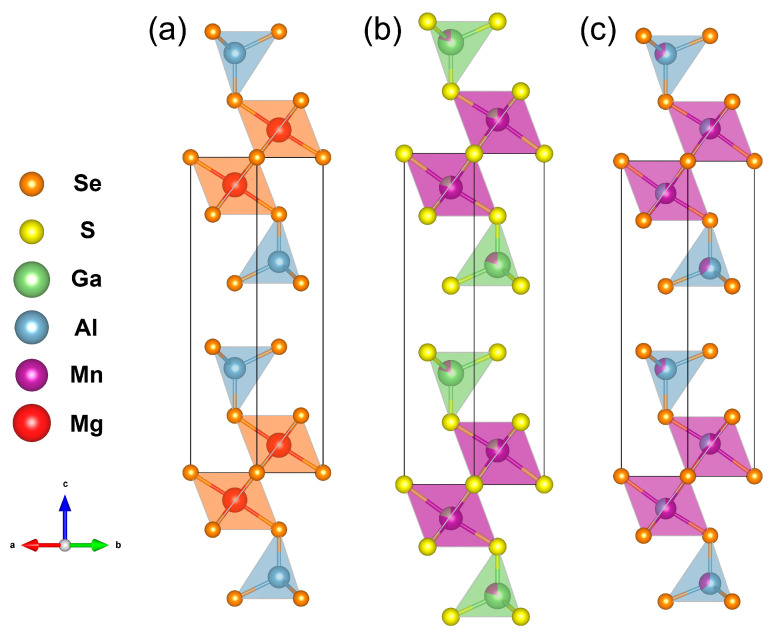
Crystal structures of (**a**) Mg_2_Al_2_Se_5_, (**b**) Mn_2_Ga_2_S_5_, and (**c**) Mn_2_Al_2_Se_5_.

The crystal structure of Mn_2_Ga_2_S_5_ was studied previously [30], but the mixed occupation of cationic positions was not considered. In this work, we have established the mixed occupancy of crystallographic positions by Mn and Ga atoms. The Mn_2_Al_2_Se_5_ compound has been prepared and investigated for the first time. The positions of atoms and selected interatomic distances of the refined crystal structures are shown in Table 1 and Table 2, respectively. Mn_2_Ga_2_S_5_ and Mn_2_Al_2_Se_5_ are isomorphous, crystallizing in the Mg_2_Al_2_Se_5_ structure type (Figure 5). Both compounds demonstrate a mixed population of cationic positions. For Mn_2_Ga_2_S_5_, the occupation of Mn^2+^ cations in the octahedral position is 79.5%, while the tetrahedral position contains 79.5% of Ga^3+^ cations. In the crystal structure of Mn_2_Al_2_Se_5_, Mn^2+^ and Al^3+^ jointly populate the octahedral position, with the ratio of 64.5%/35.5%, respectively. The assumed charge of Mn^2+^ with the 3*d*^5^ electron shell configuration in the high-spin state has the same preference and no energy difference between the octahedral and tetrahedral environment from the point of view of the crystal field theory, which may be a reason for the mixed occupation of octahedral and tetrahedral sites in the crystal structures.

### 2.3. Magnetic Properties

**Figure 6:**
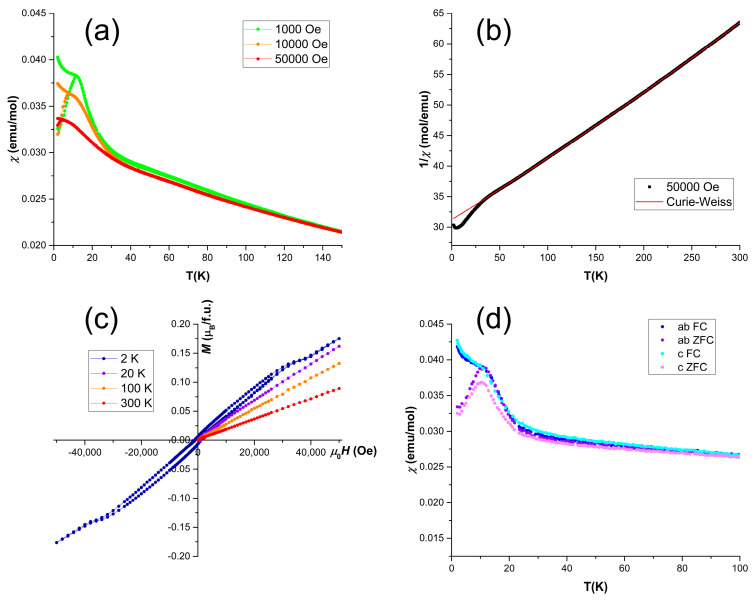
Magnetic properties of Mn_2_Ga_2_S_5_: (**a**) magnetic susceptibility of the polycrystalline sample in various magnetic fields; (**b**) Curie–Weiss fit of the high-temperature inverse susceptibility of polycrystalline Mn_2_Ga_2_S_5_; (**c**) magnetization curves measured on the oriented single crystal for H||c; (**d**) magnetic susceptibility of the oriented single crystal for H||ab and H||c.

The magnetic properties of Mn_2_Ga_2_S_5_ were investigated for polycrystalline and single-crystal samples via DC magnetization measurements (Figure 6). The temperature dependence of magnetic susceptibility demonstrates an antiferromagnetic-like transition at T_N_ = 12.7 K and high-temperature Curie–Weiss-type behavior in agreement with the previous reports [29,30]. Magnetization curves above T_N_ indicate the absence of magnetic admixtures, while the slight ferromagnetic-like hysteresis below T_N_ is presumably due to the strong frustration of magnetic moments. Furthermore, the hysteresis of magnetization at 2 K demonstrates change of the slope in the vicinity of the 3.6 T magnetic field, which may be due to the canting of magnetic moments with respect to the easy-axis direction. Approximation of the high-temperature data via the modified Curie-Weiss law yields the Weiss temperature of *θ* = −350 K and an effective magnetic moment of µ = 4.97 µ_B_. The Weiss temperature indicates a sufficiently strong exchange interaction between the magnetic centers. Furthermore, the observed large value of T_N_/|*θ*| = 28 points at a high degree of magnetic frustration in the system, which may be caused by the triangular arrangement of Mn^2+^ species between the hexagonal S layers and by the mixed occupation of octahedral and tetrahedral sites, as observed in the PXRD experiments. The effective magnetic moment is slightly lower than expected for Mn^2+^ = 5.92 μ_B_ [31]. Magnetic susceptibility of the oriented single crystal demonstrates slight anisotropy below the transition temperature, which is absent in the high-temperature paramagnetic state.

### 2.4. Morphology and Nanomaterials

**Figure 7:**
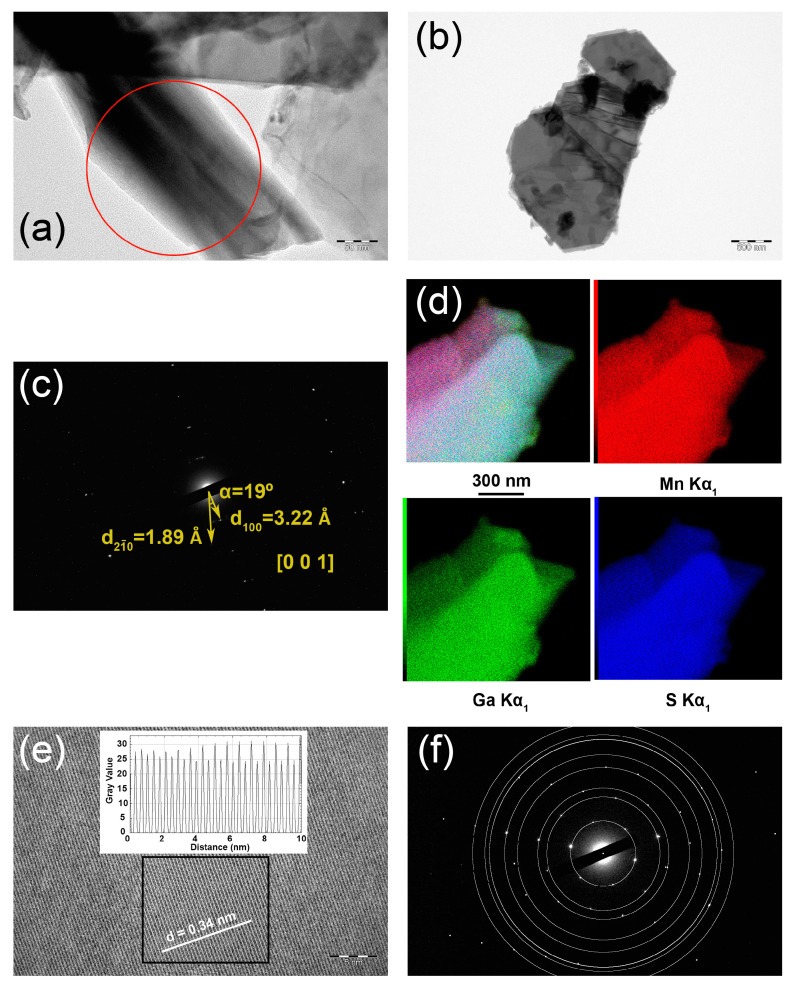
EDXS mapping and HRTEM of Mn_2_Ga_2_S_5_ nanomaterials: (**a**) typical bright-field TEM image of nanoscroll; (**b**) bright-field TEM image of a flat crystallite; (**c**) SAED pattern collected from the area, which is shown by the red circle in (**a**); (**d**) EDXS mapping; (**e**) HRTEM image of the [001] zone; the inset shows intensity profile measured along the white line; (**f**) SAED pattern taken along the [001] direction from the area shown by the black rectangle in (**e**). The white circles indicate the series of reflections with d-spacings presented in Table 4.

A polycrystalline sample of Mn_2_Ga_2_S_5_ was used to prepare nanomaterials via exfoliation in a non-polar solvent. Two types of crystallites are observed using high-resolution transmission electron microscopy (HRTEM): nanoscrolls, which are formed as a result of bending of atomically thin flakes (Figure 7a), and microflakes with a thickness of 10–100 nm (Figure 7b). EDXS mapping confirms the uniform distribution of Mn, Ga, and S species in the studied crystallites. The selected area electron diffraction (SAED) pattern collected from the nanoscrolls (Figure 7c) shows the splitting of the 100 reflections as a result of the material bending during the formation of nanoscrolls. Figure 7e shows the [001] zone found in the central part of a flake. A HRTEM image with atomic resolution clearly indicates the d_100_ spacing of the electron density. Furthermore, the SAED pattern collected from the [001] zone can be indexed using the unit cell parameters of Mn_2_Ga_2_S_5_ obtained from the PXRD experiment. The SAED- and PXRD-based values of d-spacing are in perfect agreement, confirming the close-packing motif of the crystal structure (Table 4).

**Figure 8:**
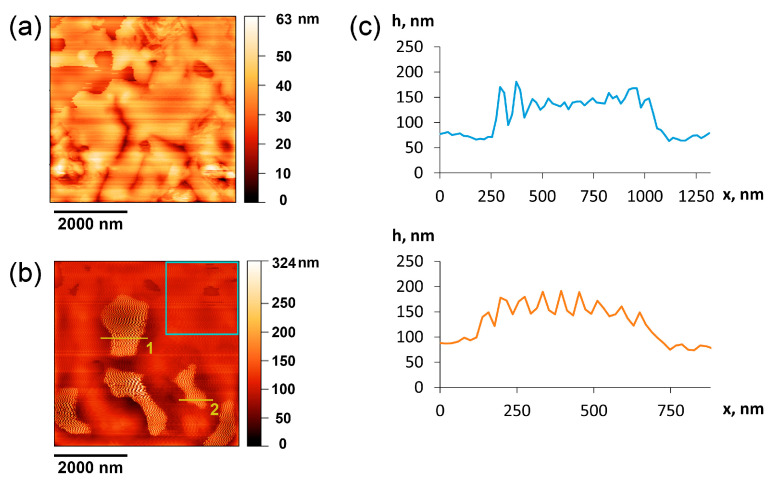
AFM topography image of (**a**) empty glass substrate and (**b**) deposited Mn_2_Ga_2_S_5_ particles. (**c**) Height profile along the yellow lines (1—top; 2—bottom).

The study of substrates with deposited particles of Mn_2_Ga_2_S_5_ was performed using atomic force microscopy (AFM). To confirm the presence of particles on the surface, we calculated the roughness of an empty glass substrate surface (Figure 8a) and the surface area of 2 × 2 µm^2^ (Figure 8b, blue frame) after deposition, where no particles are present. The root mean squared roughness value for the 2 × 2 µm^2^ fragment of the area on raw glass was 6–8 nm, and it was 8.2 nm for the empty area of the sample, with a total roughness of the entire sample area of 30.5 nm. These data show the appearance of new objects on the surface. At the micron scale, it was possible to observe particles, less than 100 nm thick, with a flat surface. These particles possess a highly-oriented flat surface, which is visible in the yellow–dark contrast in Figure 8b. The oscillations are shown more clearly in the line profiles of the AFM signal (Figure 8c). The observed type of distortion persists while changing the scanning speed within 0.2–1 rows per second and applying different forces of pressure of the cantilever onto the surface. These oscillations are probably associated with weak adhesion of the particles to the glass surface; so, particles can be easily deformed by the cantilever and respond to it as a non-rigid surface. Thus, the line profiles indicate a variation in the visible thickness of Mn_2_Ga_2_S_5_ particles that does not exceed 70 nm.

## 3. Materials and Methods

For the synthesis of Mn_2_Ga_2_S_5_, Mn (plates, 99%, Sigma-Aldrich, St. Louis, MO, USA), Ga (lump, 99.99%, Sigma-Aldrich, United States), and S (powder, 99.98%, Sigma-Aldrich, St. Louis, MO, USA) were used as precursors. The binary precursors MnSe and Al_2_Se_3_ were synthesized from Mn (described earlier), Al (granules, 99.999% Merck, Rahway, NJ, USA), and Se (granules, 99.999%, Sigma-Aldrich, St. Louis, MO, USA). All operations with samples were performed in an argon-filled glove box (Spectro-systems, p(H_2_O, O_2_) < 1 ppm). All precursors were weighed in the stoichiometric ratio with accuracy of 10^−4^ g. Before annealing, all substances were placed in a quartz ampule, evacuated to a residual gas pressure of 5 × 10^−3^ mbar, and flame-sealed. The Mn_2_Ga_2_S_5_ sample was annealed twice at 1173 K for 5 days with intermediate grinding. MnSe and Al_2_Se_3_ binary selenides were obtained by annealing the elements at 973 K for 5 days. After checking their purity by PXRD, MnSe and Al_2_Se_3_ were mixed in the stoichiometric ratio and pressed into a pellet with a diameter of 6 mm at a pressure of 1200 kgf/cm^2^. Synthesis of Mn_2_Al_2_Se_5_ was performed by annealing the pellet at 1123 K for 5 days. For the growth of single crystals, Mn_2_Ga_2_S_5_ powder (total mass of 0.5 g) was placed in a quartz ampule and, after adding 15 mg of HgI_2_, evacuated and flame-sealed. The ampule was placed in a tube furnace with a temperature of the hot zone T_1_ = 1273 K and the cold zone T_2_ = 1173 K and annealed for 10 days. The resulting crystals were observed in the cold zone.

A phase composition study and crystal structure refinements were performed via PXRD using data obtained on a Huber G670 Guinier camera (Cu Kα_1_ radiation, Ge_111_ monochromator, image plate detector). The samples were enclosed between two mylar films and fixed in the sample holder in an argon-filled glove box. Crystal structure was refined using the Jana2006 software package. CCDC 2344029 and 2344030 deposition numbers can be used to request the structural data. Chemical stability was studied using a Bruker D8 Advance powder diffractometer (Cu X-ray source, no monochromator, LYNXEYE detector). The experiment was performed in the Bragg–Brentano geometry; thus, the preferred orientation of crystallites along the [001] direction is present on the registered patterns (Figure 2). The measurement and refinement details, parameters of atomic positions, and selected interatomic distances are given in Table 1, Table 2 and Table 3, respectively. According to the Rietveld analysis, the structural *R*-factors of *R_obs_* = 7.8% for Mn_2_Ga_2_S_5_ and 4.4% for Mn_2_Al_2_Se_5_ indicate the validities of structural models of 92.2% and 95.6%, respectively.

Images of single crystals and mapping and analysis of elemental compositions were obtained using scanning electron microscopes JSM JEOL6490-LV and Carl Zeiss Leo SUPRA 50 VP with energy-dispersive X-ray detectors INCA at accelerating voltage of 20 kV. The polycrystalline sample of Mn_2_Ga_2_S_5_ was studied using HRTEM on a JEOL JEM-2100 UHR at accelerating voltage of 200 kV. Before analysis, suspension of the sample in heptane was dropped to a copper grid and treated with plasma. SAED patterns were calibrated using a standard gold sample. Images from the HRTEM were processed using the ImageJ package [32].

Glass substrates were used to deposit nanomaterials. The films were examined via AFM, which was performed using NTEGRA Aura (NT-MDT, Moscow, Russia) in a semicontact mode.

The magnetization of single-crystal and polycrystalline samples was measured using a Magnetic Properties Measurement System (MPMS-XL5 SQUID, Quantum Design, San Diego, CA, USA). The measurements were performed in zero-field cooling (ZFC) and field cooling (FC) conditions. Temperature dependencies were measured from 2 K to 300 K in magnetic fields of 0.01, 0.1, 1, and 5 T, and field dependences were measured at temperatures 2, 6, 10, 20, 50, 100, 200, and 300 K when scanning the magnetic field from −5 to 5 T.

## 4. Conclusions

In this work, we report on the synthesis, structure, and properties of two chalcogenides, Mn_2_Ga_2_S_5_ and Mn_2_Al_2_Se_5_, belonging to the family of layered van der Waals compounds. For the latter compound, which is hygroscopic, only synthesis and crystal structure determination are reported, whereas for the former chalcogenide, the entire investigation, including magnetic properties evaluation and fabrication of nanomaterials via exfoliation, was performed. Both chalcogenides exhibit a mixed population of cationic positions, have a van der Waals gap in their crystal structures, and display typical features of layered compounds; in particular, texturing and preferential orientation of crystallites perpendicular to the crystallographic *c*-axis were observed via powder X-ray diffraction and selected area electron diffraction. Magnetic measurements reveal a sufficiently strong exchange interaction between the magnetic centers and a high degree of magnetic frustration in Mn_2_Ga_2_S_5_, which may be caused by the triangular arrangement of Mn^2+^ cations and the mixed population of octahedral and tetrahedral sites by manganese and gallium. Using liquid exfoliation in a non-polar solvent under ultrasonic treatment, it was possible to obtain Mn_2_Ga_2_S_5_ nanoflakes and nanoscrolls. Further studies of the electronic and magnetic properties of these low-dimensional materials are highly desirable. They can be utilized in the design of heterostructures with further application in spintronics.

## Figures and Tables

**Table 1:** Crystallographic data and experimental details for Mn_2_Ga_2_S_5_ and Mn_2_Al_2_Se_5._

Parameter	Value	
composition	Mn_2_Ga_2_S_5_	Mn_2_Al_2_Se_5_
formula weight (g/mol)	409.647	558.639
diffractometer	Huber G670	
detector	image plate	
radiation	Cu K*α*_1_	
wavelength (Å)	1.5406	
crystal system	trigonal	
space group	*P*$\bar{3}$*m*1	
unit cell parameters		
*a* (Å)	3.71694(3)	3.89660(4)
*c* (Å)	15.2237(2)	15.9912(2)
*V* (Å^3^)	182.146(2)	210.272(4)
temperature (K)	293	
*ρ*_calc_ (g/cm^3^)	3.73	4.41
μ (cm^−1^)	48.55	50.48
2*θ* range (deg)	3–100.3	3–100.3
*R*_p_	0.0255	0.0207
*wR*_p_	0.0368	0.0276
*R*_obs_	0.0776	0.0439
*wR*_obs_	0.0951	0.0535
*R*_all_	0.0857	0.0452
*wR*_all_	0.0952	0.0538
GOF	2.84	1.66
parameters	29	32
constraints	3	5
residual peaks (e^−^/Å^3^)	2.78/−2.06	1.37/−1.36

**Table 2:** Atomic parameters for the crystal structures of Mn_2_Ga_2_S_5_ (a) and Mn_2_Al_2_Se_5_ (b).

**(a)**	**Mn_2_Ga_2_S_5_**					
**Label**	**Symmetry**	***x***	***y***	***z***	**Occupancy**	***U*_iso_ (Å^2^)**
Mn1	3*m*	1/3	2/3	0.1024(1)	0.791(5)Mn + 0.209Ga	0.0194(6)
Ga1	3*m*	1/3	2/3	0.6635(1)	0.791Ga + 0.209Mn	0.0188(5)
S1	$\bar{3}$ *m*	0	0	0	1	0.009(1)
S2	3*m*	1/3	2/3	0.3989(2)	1	0.0049(6)
S3	3*m*	1/3	2/3	0.8115(2)	1	0.0277(9)
**(b)**	**Mn_2_Al_2_Se_5_**					
**Label**	**Symmetry**	***x***	***y***	***z***	**Occupancy**	***U*_iso_(Å^2^)**
Mn1	3*m*	1/3	2/3	0.10275(7)	0.645(1)Mn + 0.355Al	0.0203(3)
Al1	3*m*	1/3	2/3	0.65970(4)	0.645Al + 0.355Mn	0.0260(3)
Se1	$\bar{3}$ *m*	0	0	0	1	0.0382(6)
Se2	3*m*	1/3	2/3	0.39710(6)	1	0.0138(5)
Se3	3*m*	1/3	2/3	0.81149(6)	1	0.0291(6)

**Table 3:** Interatomic distances for cations in the crystal structures of Mn_2_Ga_2_S_5_ (a) and Mn_2_Al_2_Se_5_ (b).

**(a)**	**Mn_2_Ga_2_S_5_**	
**Central Atom**	**Neighbor Atom**	**Distance (Å)**
Mn1	S1 (×3)	2.652(1)
	S3 (×3)	2.515(2)
Ga1	S2 (×3)	2.347(1)
	S3 (×1)	2.253(3)
**(b)**	**Mn_2_Al_2_Se_5_**	
**Central Atom**	**Neighbor Atom**	**Distance (Å)**
Mn1	Se1 (×3)	2.7858(6)
	Se3 (×3)	2.6347(7)
Al1	Se2 (×3)	2.4261(4)
	Se3 (×1)	2.427(1)

**Table 4:** Comparison of d-spacing values from PXRD and SAED.

d_SAED_, Å	d_XRD_, Å	h	k	l
3.25	3.22	1	0	0
1.87	1.86	2	$\bar{1}$	0
1.61	1.61	2	0	0
1.23	1.22	3	$\bar{1}$	0
1.07	1.07	3	0	0
0.93	0.93	4	$\bar{2}$	0
0.89	0.89	4	$\bar{1}$	0
0.82	0.80	4	0	0

## Data Availability

Dataset available on request from the authors.
